# Supplementary material for: Single-cell RNA sequencing of peripheral blood mononuclear cells from acute Kawasaki disease patients
Source: Nat Commun. 2021 Sep 14;12:5444. doi: 10.1038/s41467-021-25771-5 (PMC8440575; doi:10.1038/s41467-021-25771-5)
Supplement: Supplementary file 1 — Supplementary Information [file 41467_2021_25771_MOESM1_ESM.pdf]

Supplementary Figures and Tables

Single-cell RNA sequencing of peripheral blood mononuclear cells from acute Kawasaki disease patients

Zhen Wang, Lijian Xie, Guohui Ding, Sirui Song, Liqin Chen, Guang Li, Min Xia, Dingding Han, Yue Zheng, Jia Liu, Tingting Xiao, Hong Zhang, Yujuan Huang, Yixue Li, Min Huang

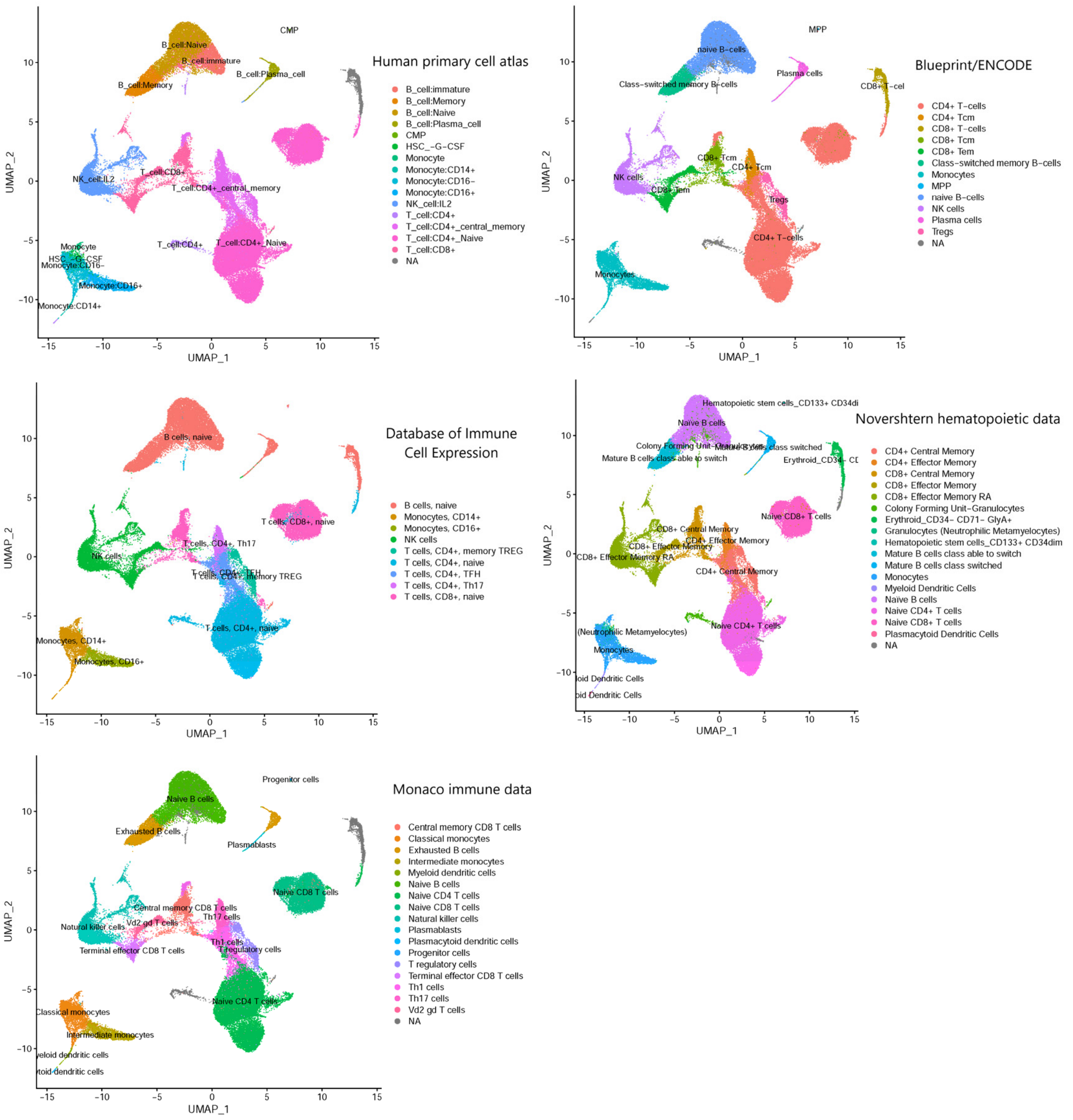

Supplementary Figure 1. Annotations of cell clusters with SingleR. Five recommended reference transcriptomic datasets were used, including the human primary cell atlas, Blueprint/ENCODE, Database of Immune Cell Expression, Novershtern hematopoietic data and Monaco immune data.

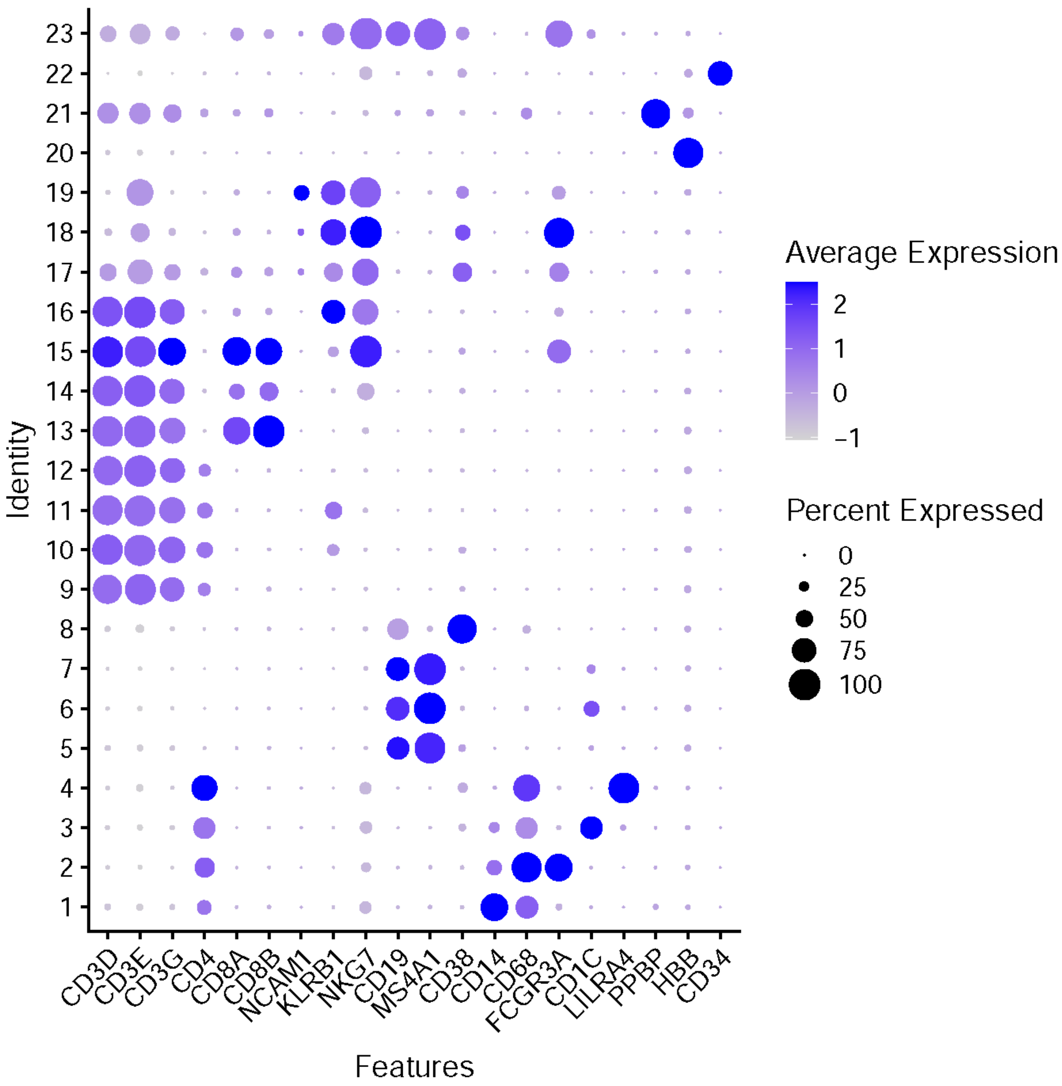

Supplementary Figure 2. Expression of marker genes for major PBMC compartments. Dot plot depicting average expression and percentage of expressing cells in each cell cluster. The cell cluster identity is the same as Figure 1.

a

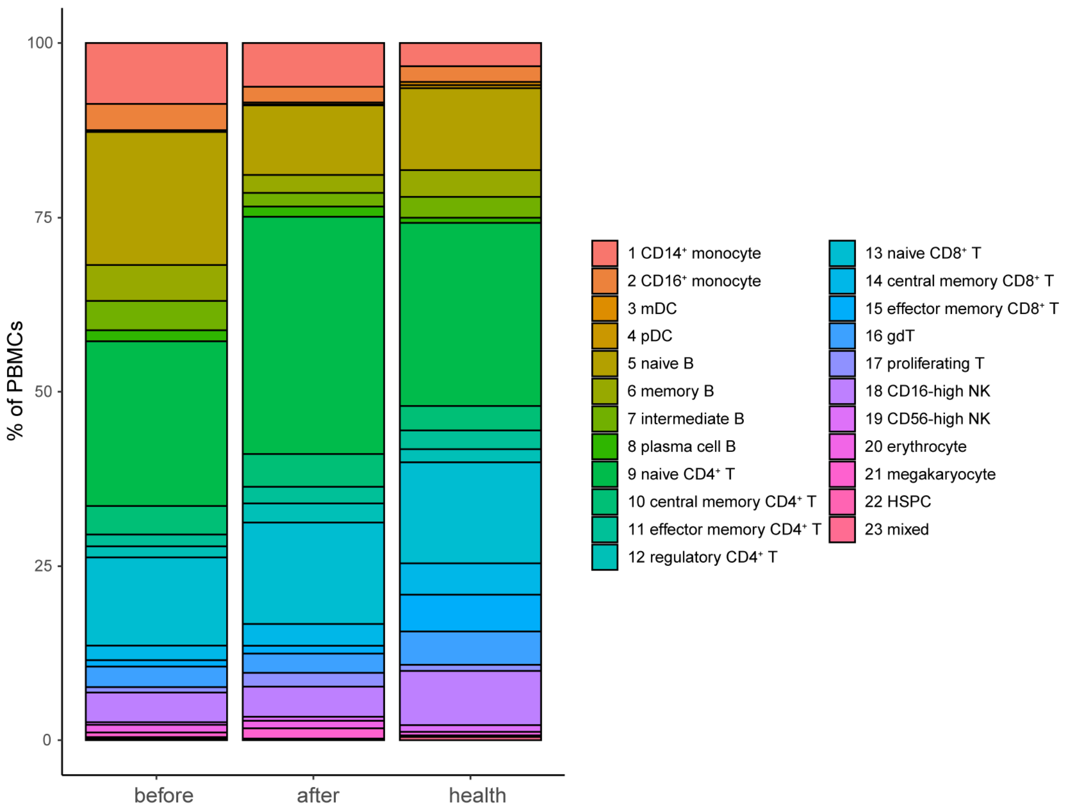

b

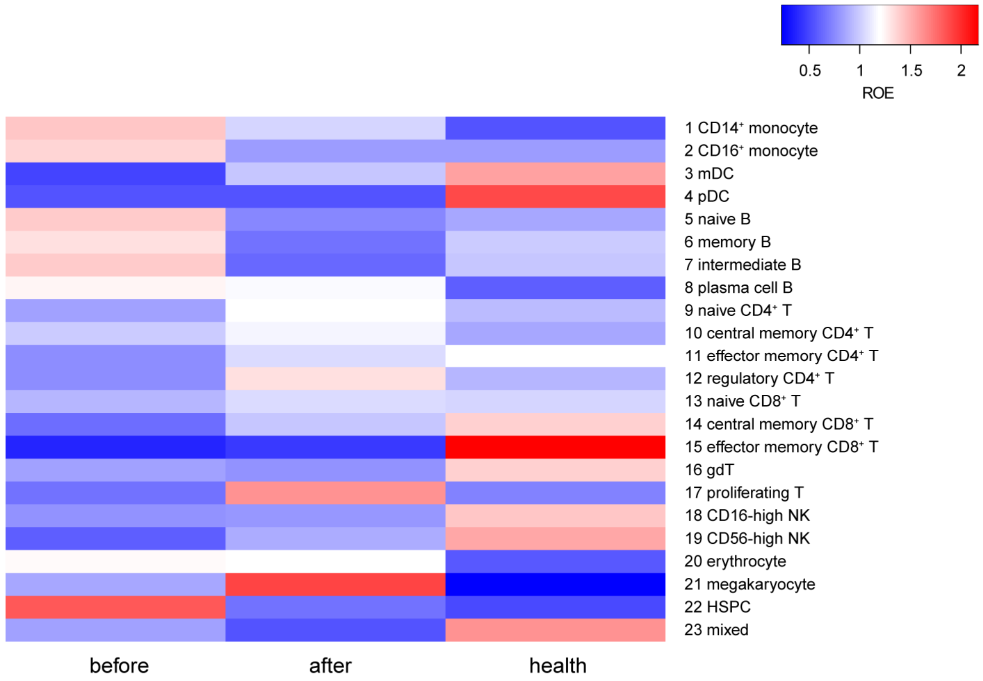

Supplementary Figure 3. Cell type abundance in pooled samples. a Percentage of cell types under each condition. **b** Ratio of observed to expected (ROE) percentage across conditions. Source data are provided in the Source Data file.

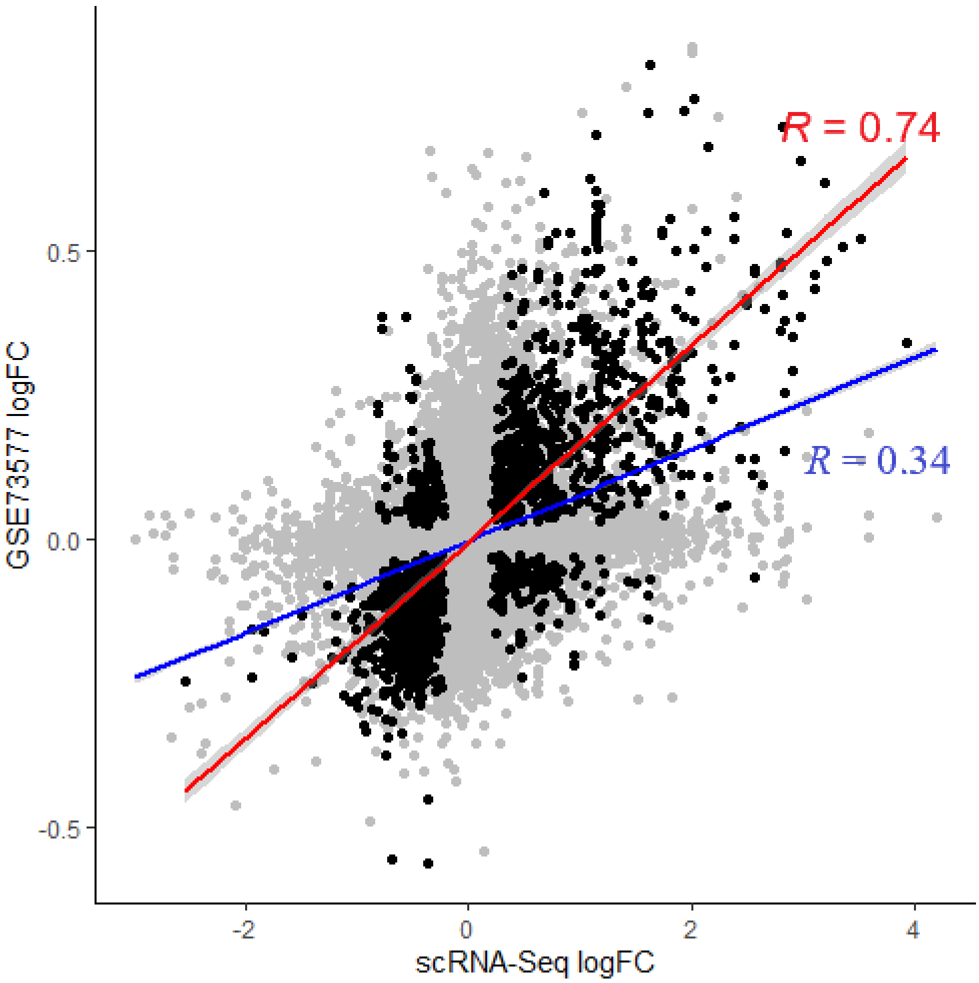

Supplementary Figure 4. Comparison of log-fold change (logFC) between scRNA-seq and GSE73577. Black points indicate DEGs (FDR < 0.05) across PBMCs and gray points indicate other genes. Red and blue line indicate Pearson's *R* for DEGs and all genes, respectively (both *P*-values < 2.2×10^{-16}, two-sided Pearson's test). Source data are provided in the Source Data file.

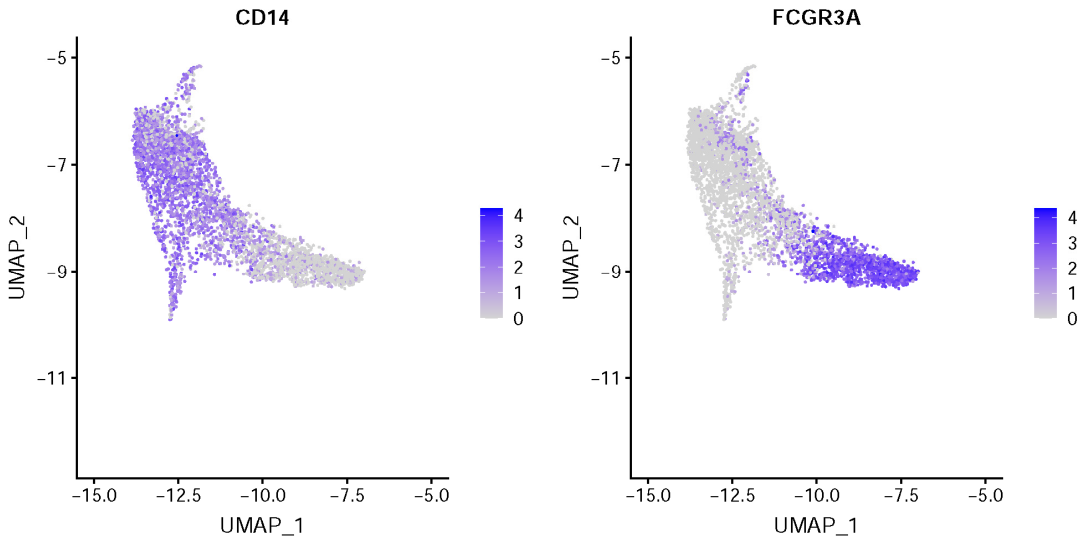

Supplementary Figure 5. Marker genes for monocyte subsets. The cells are colored based on the normalized expression of marker genes.

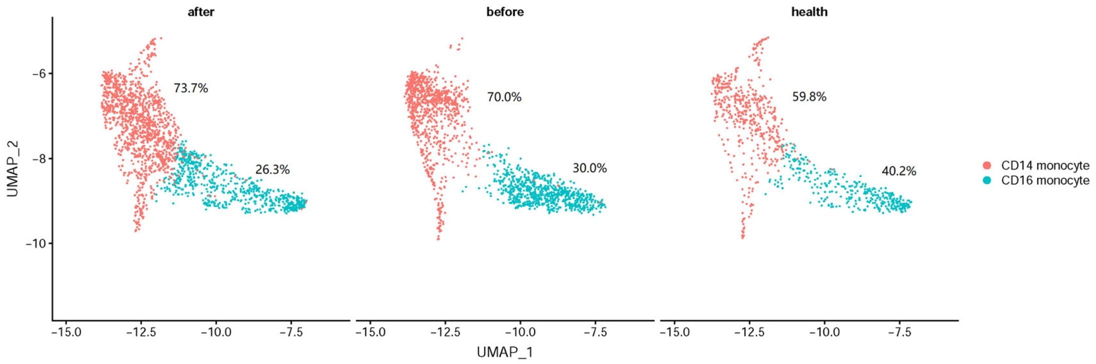

Supplementary Figure 6. Proportion of monocyte subsets separated by conditions.

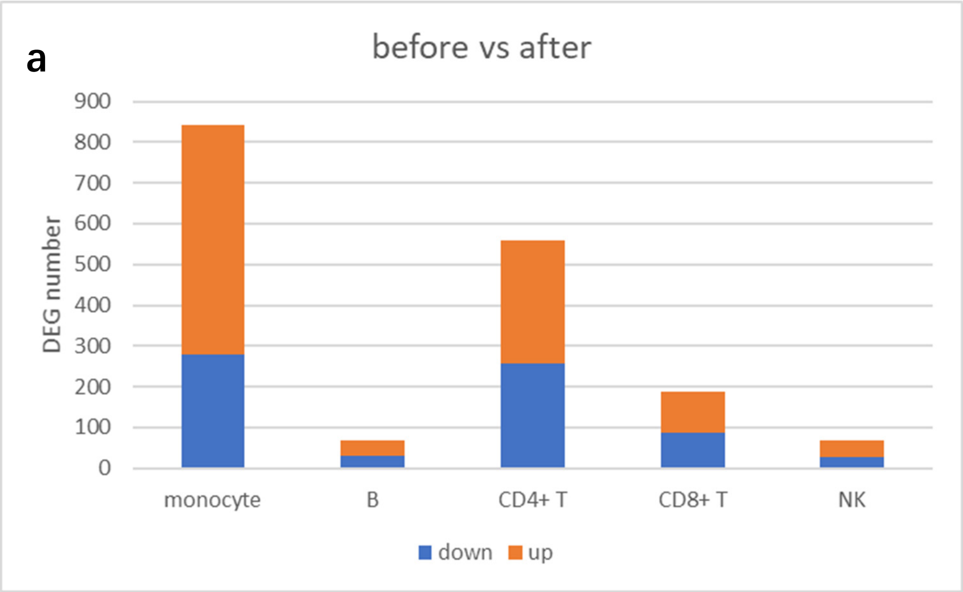

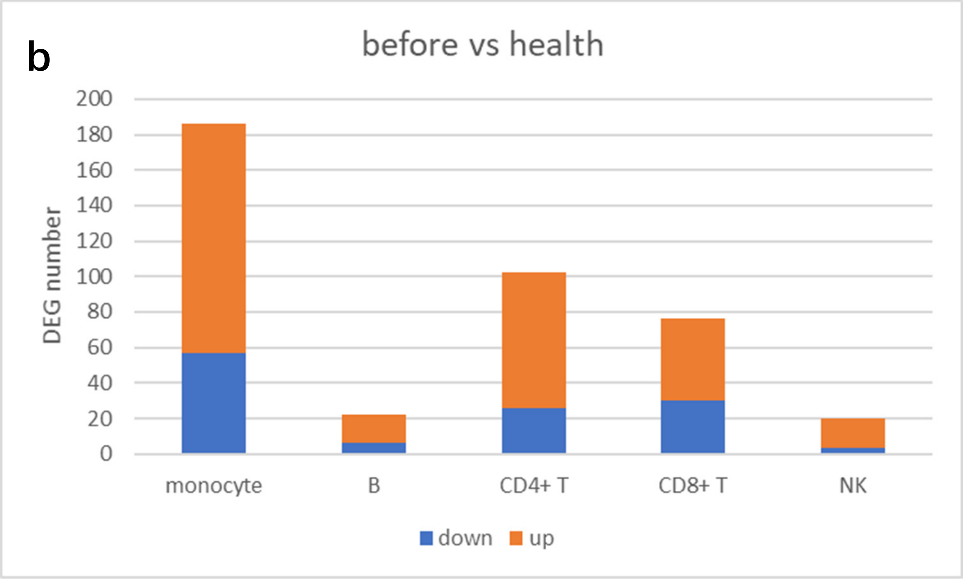

Supplementary Figure 7. Number of DEGs (FDR < 0.05) for each cell compartment. a Comparison between pre- and post-treatment samples. **b** Comparison between pre-treatment samples and healthy controls. Source data are provided in the Source Data file.

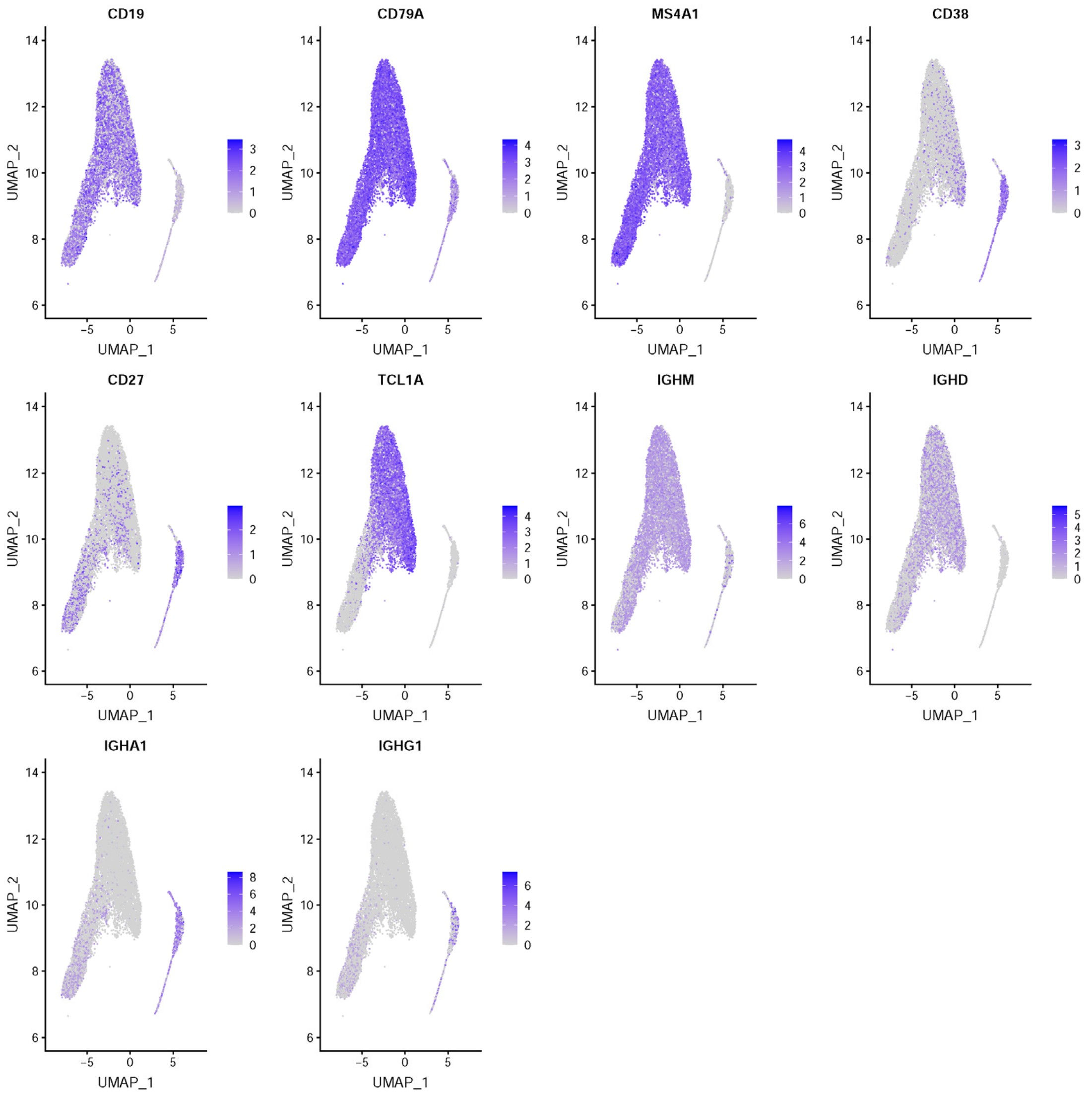

Supplementary Figure 8. Marker genes for B cell subsets. The cells are colored based on the normalized expression of marker genes.

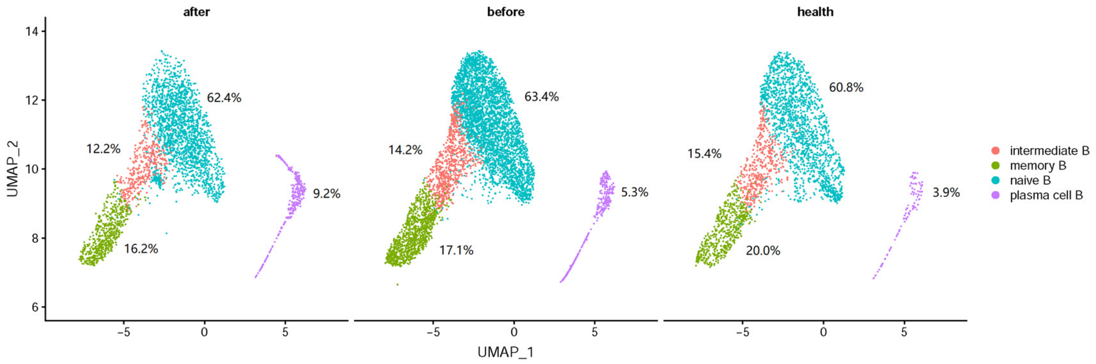

Supplementary Figure 9. Proportion of B-cell subsets separated by conditions.

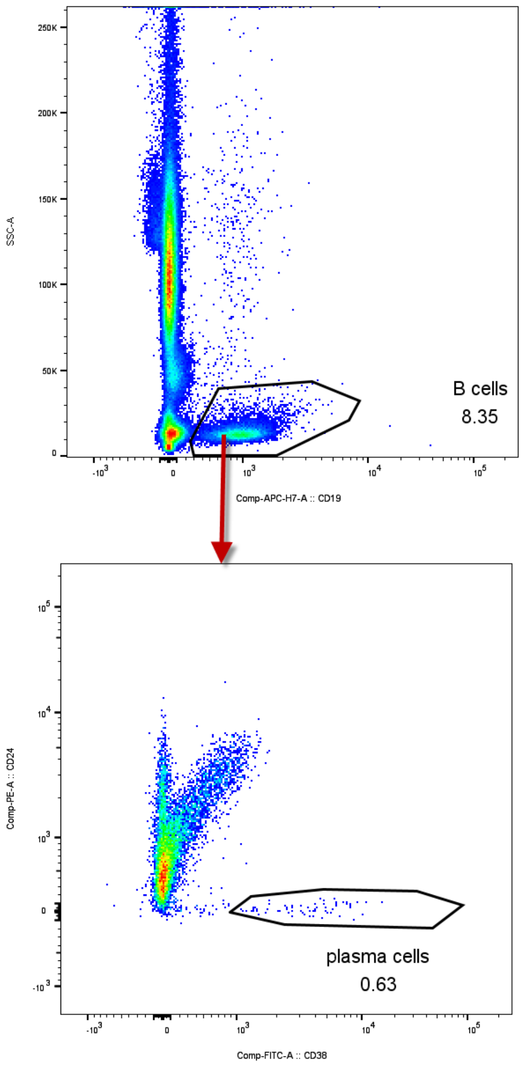

Supplementary Figure 10. Flow cytometric analysis of plasma cells. Cell markers used were $CD19^{+}$ (B cells) and $CD24^{-}CD38^{++}$ (plasma cells).

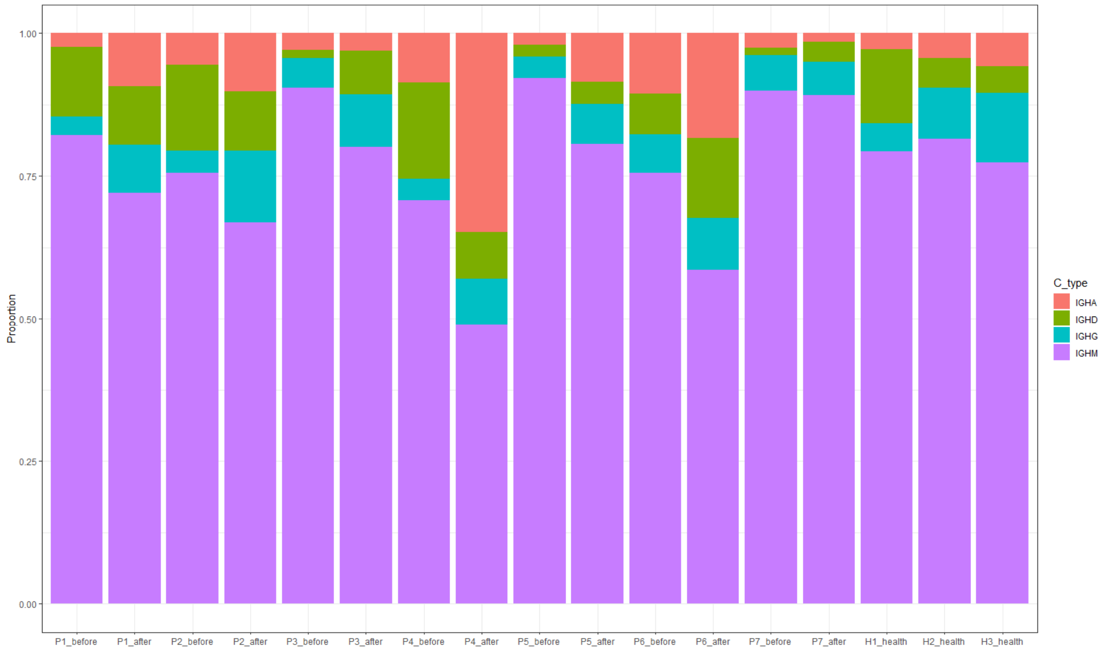

Supplementary Figure 11. Proportion of IGH isotypes in each sample. Source data are provided in the Source Data file.

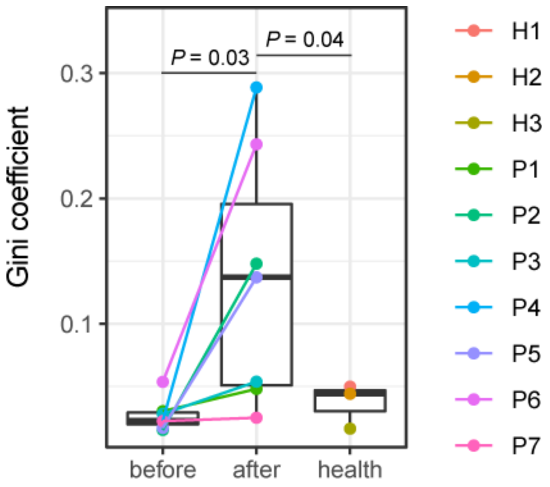

Supplementary Figure 12. Gini coefficient of the BCR repertoires. *P*-values were calculated with two-sided *t*-test (KD patients $n = 7$, healthy controls $n = 3$). The middle line of the boxplot is the median, the lower and upper hinges correspond to the first and third quartiles, and the whiskers extend from the hinge to the farthest data point within a maximum of 1.5 × interquartile range. Source data are provided in the Source Data file.

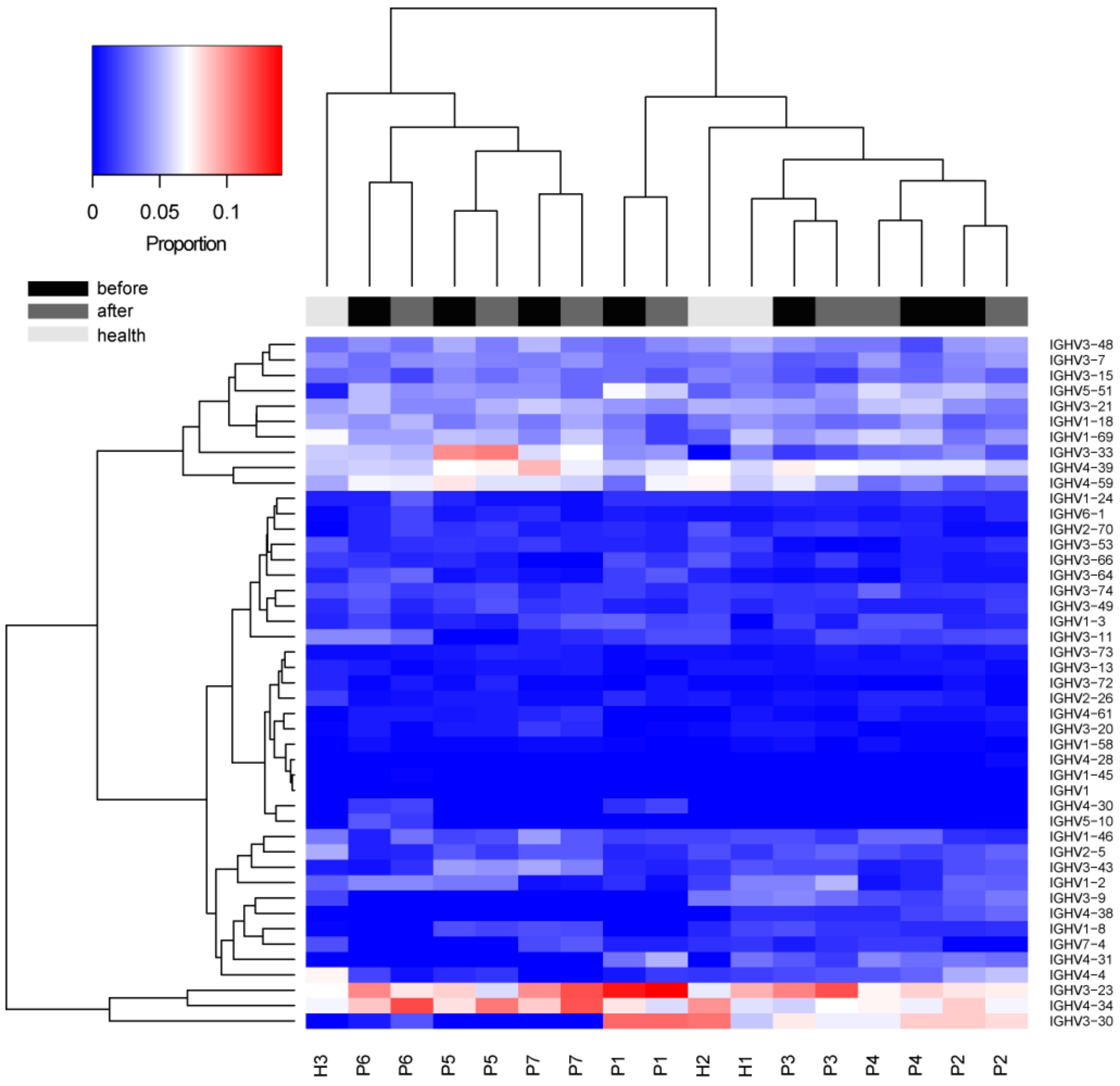

Supplementary Figure 13. IGHV gene usage in the BCR repertoires. Heat map represents proportion of IGHV genes across samples. Source data are provided in the Source Data file.

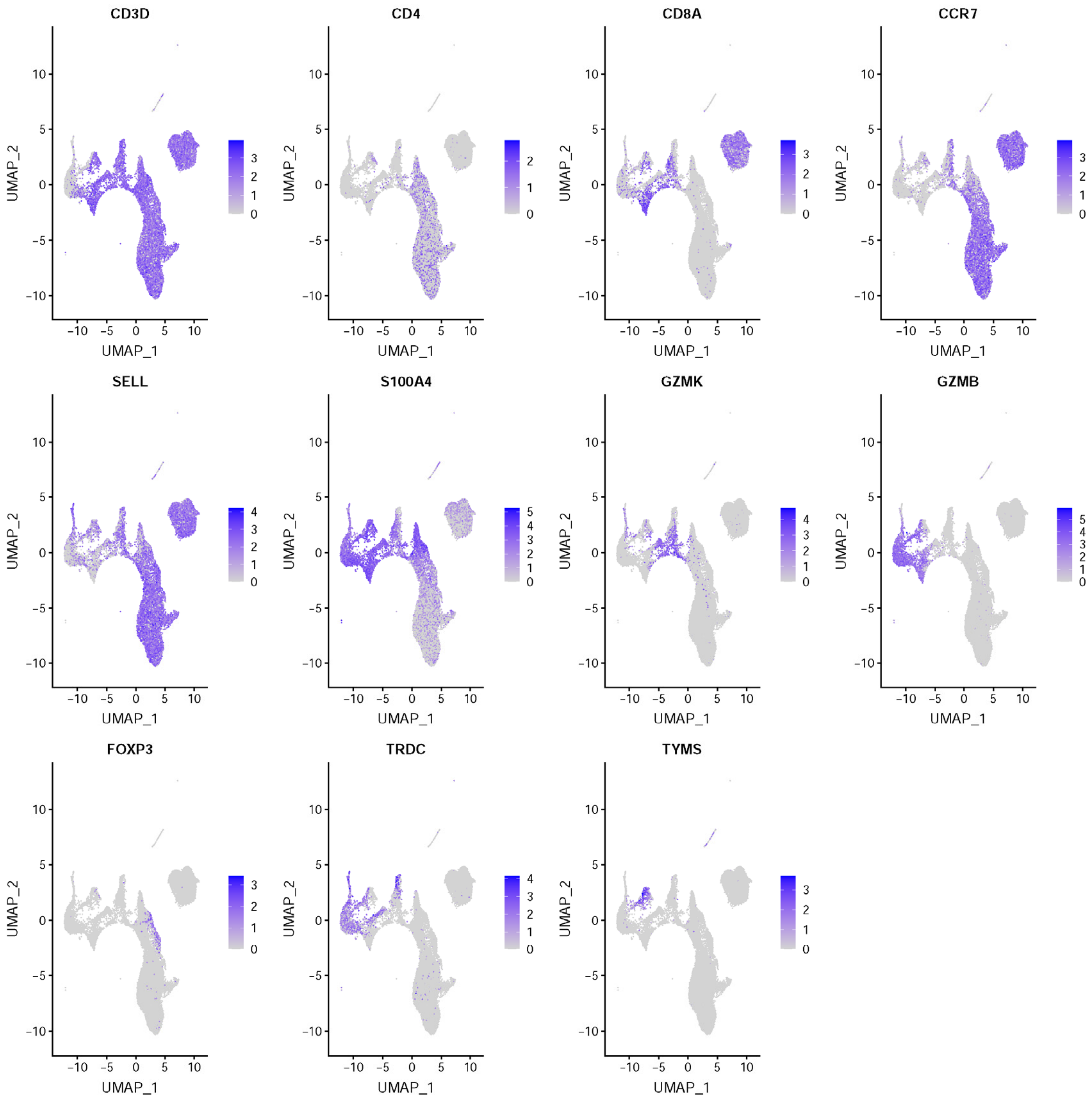

Supplementary Figure 14. Marker genes for T cell subsets. The cells are colored based on the normalized expression of marker genes.

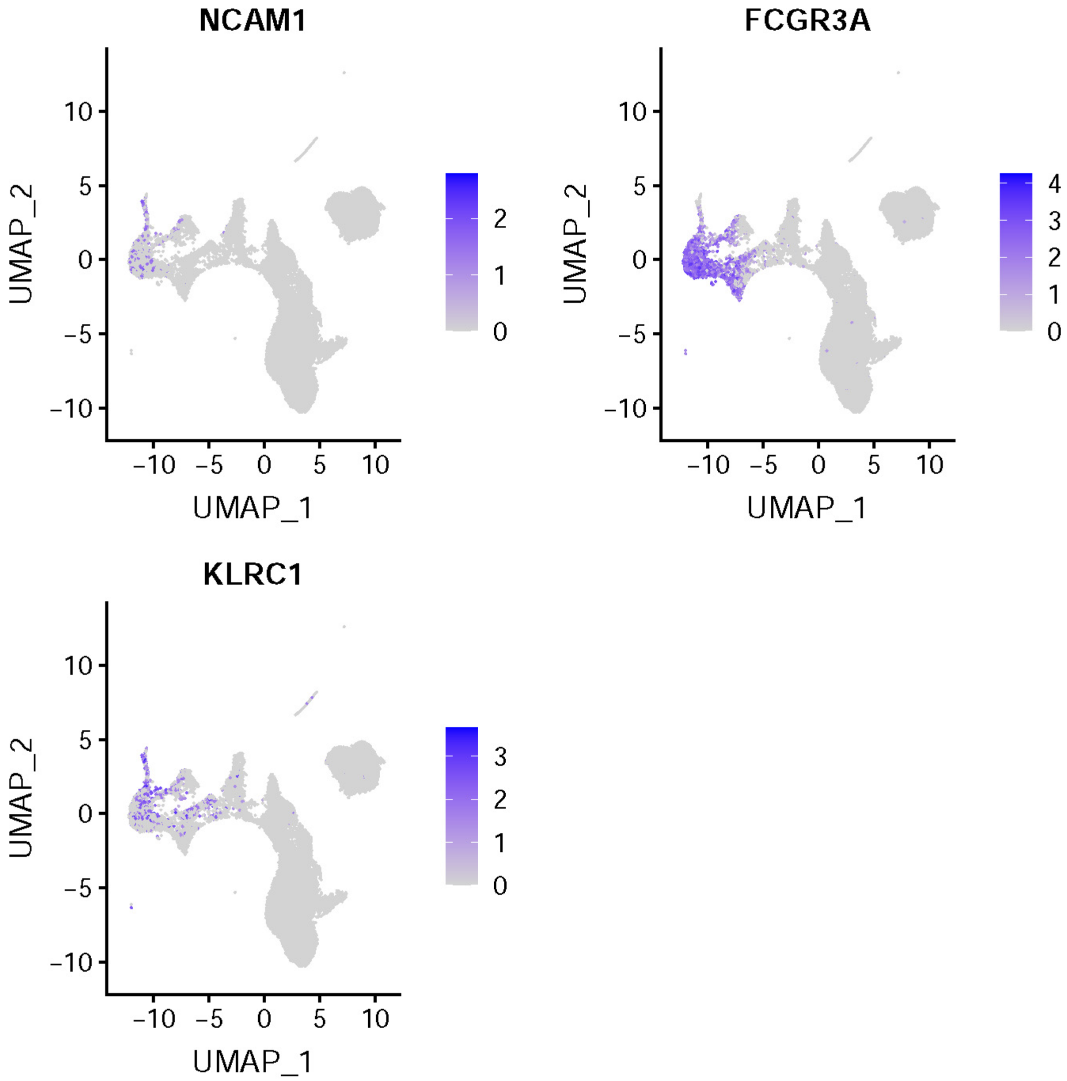

Supplementary Figure 15. Marker genes for NK cell subsets. The cells are colored based on the normalized expression of marker genes.

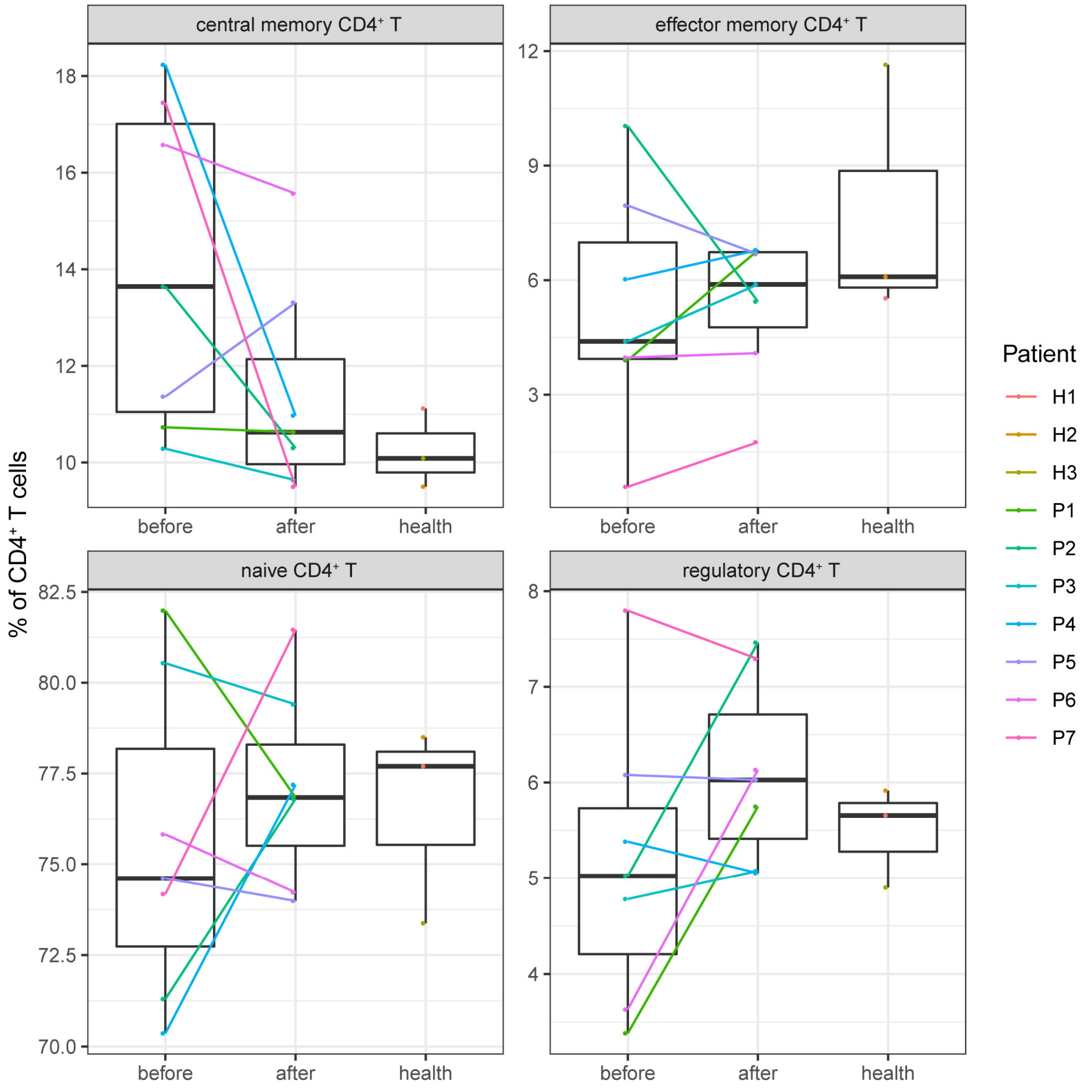

Supplementary Figure 16. Percentage of CD4$^+$ T cell subsets across conditions. KD patients n = 7, healthy controls n = 3. Data are represented as boxplots where the middle line is the median, the lower and upper hinges correspond to the first and third quartiles, and the whiskers extend from the hinge to the farthest data point within a maximum of 1.5 × interquartile range. Source data are provided in the Source Data file.

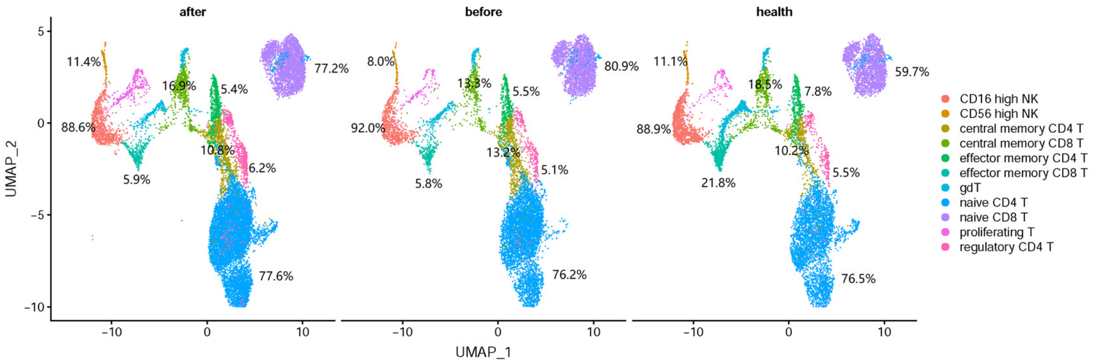

Supplementary Figure 17. Proportion of T and NK subsets separated by conditions. The proportions were calculated for $CD4^+$ T, $CD8^+$ T and NK cells, respectively.

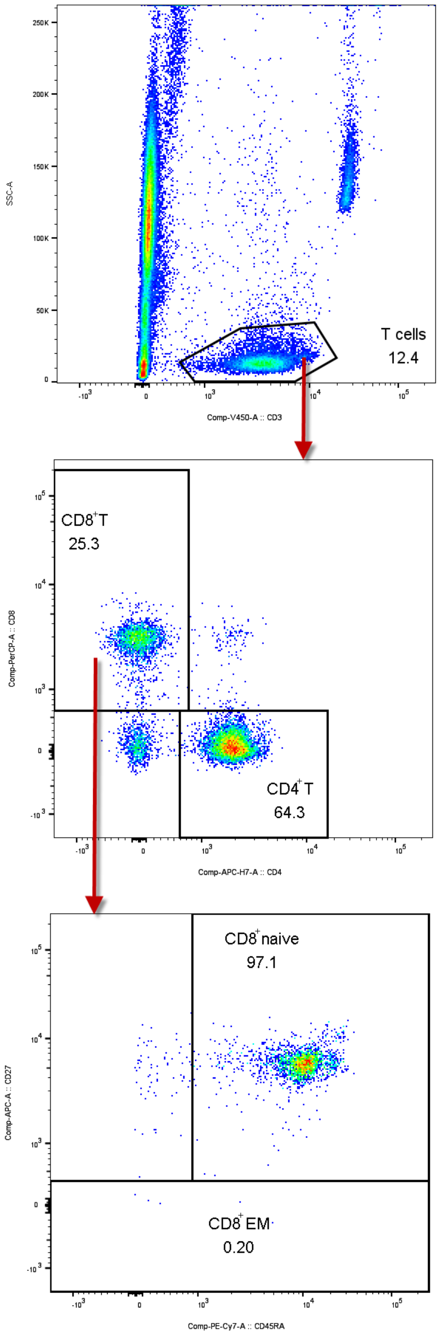

Supplementary Figure 18. Flow cytometric analysis of CD8$^+$ T cell subsets. Cell markers used were CD3$^+$ (T cells), CD8$^+$CD4$^-$ (CD8$^+$ T cells), CD27$^+$CD45RA$^+$ (naive T cells) and CD27$^-$ (effector memory [EM] T cells).

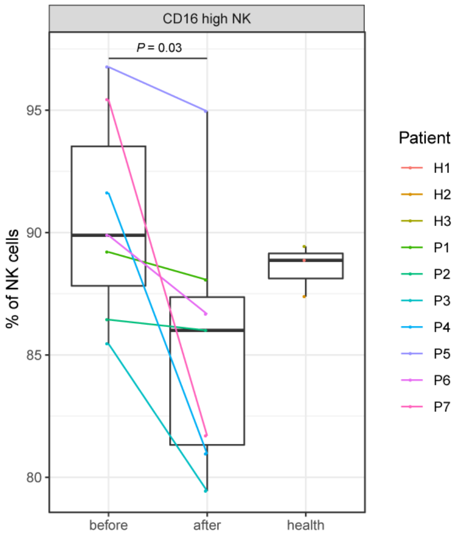

Supplementary Figure 19. Percentage of NK cell subsets across conditions. *P*-values were calculated with two-sided *t*-test (KD patients n = 7, healthy controls n = 3). The middle line of the boxplot is the median, the lower and upper hinges correspond to the first and third quartiles, and the whiskers extend from the hinge to the farthest data point within a maximum of 1.5 × interquartile range. Source data are provided in the Source Data file.

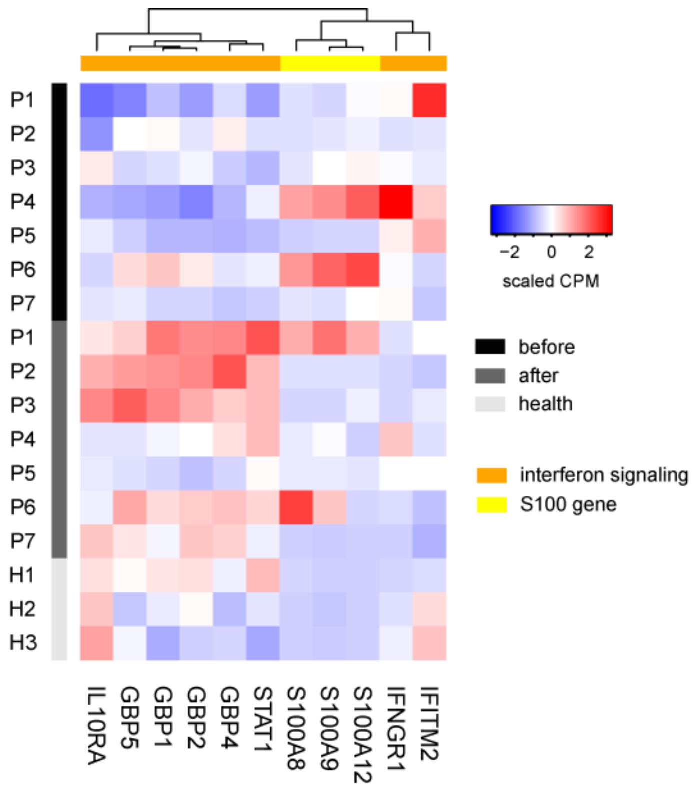

Supplementary Figure 20. Heat map of DEGs with functional enrichment in NK cells. CPM, counts per million mapped reads. Source data are provided in the Source Data file.

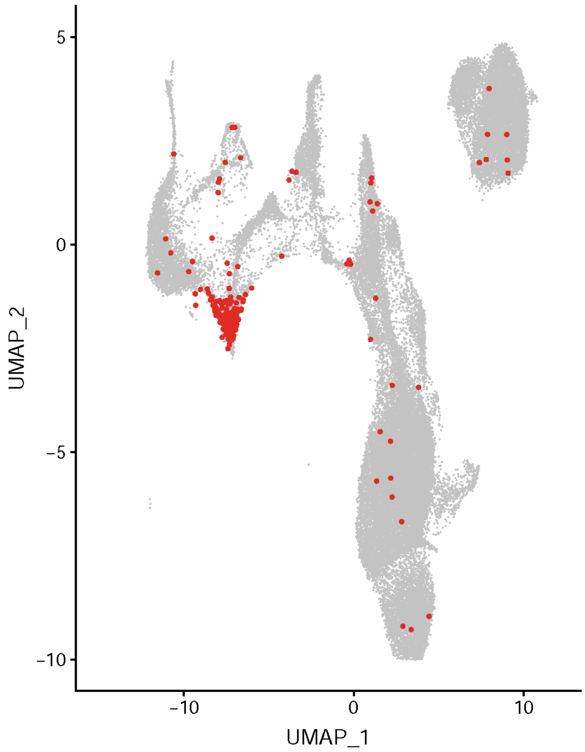

Supplementary Figure 21. T cells with clonal TCRs. T cells with clonotype size ⩾ 3 are colored in red.

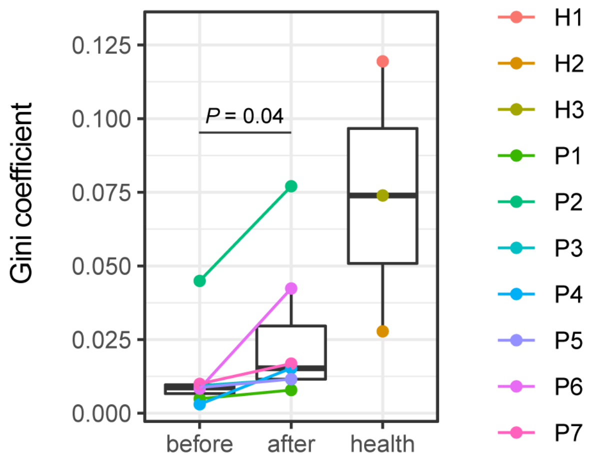

Supplementary Figure 22. Gini coefficient of the TCR repertoires. *P*-values were calculated with two-sided *t*-test (KD patients *n* = 7, healthy controls *n* = 3). The middle line of the boxplot is the median, the lower and upper hinges correspond to the first and third quartiles, and the whiskers extend from the hinge to the farthest data point within a maximum of 1.5 × interquartile range. Source data are provided in the Source Data file.

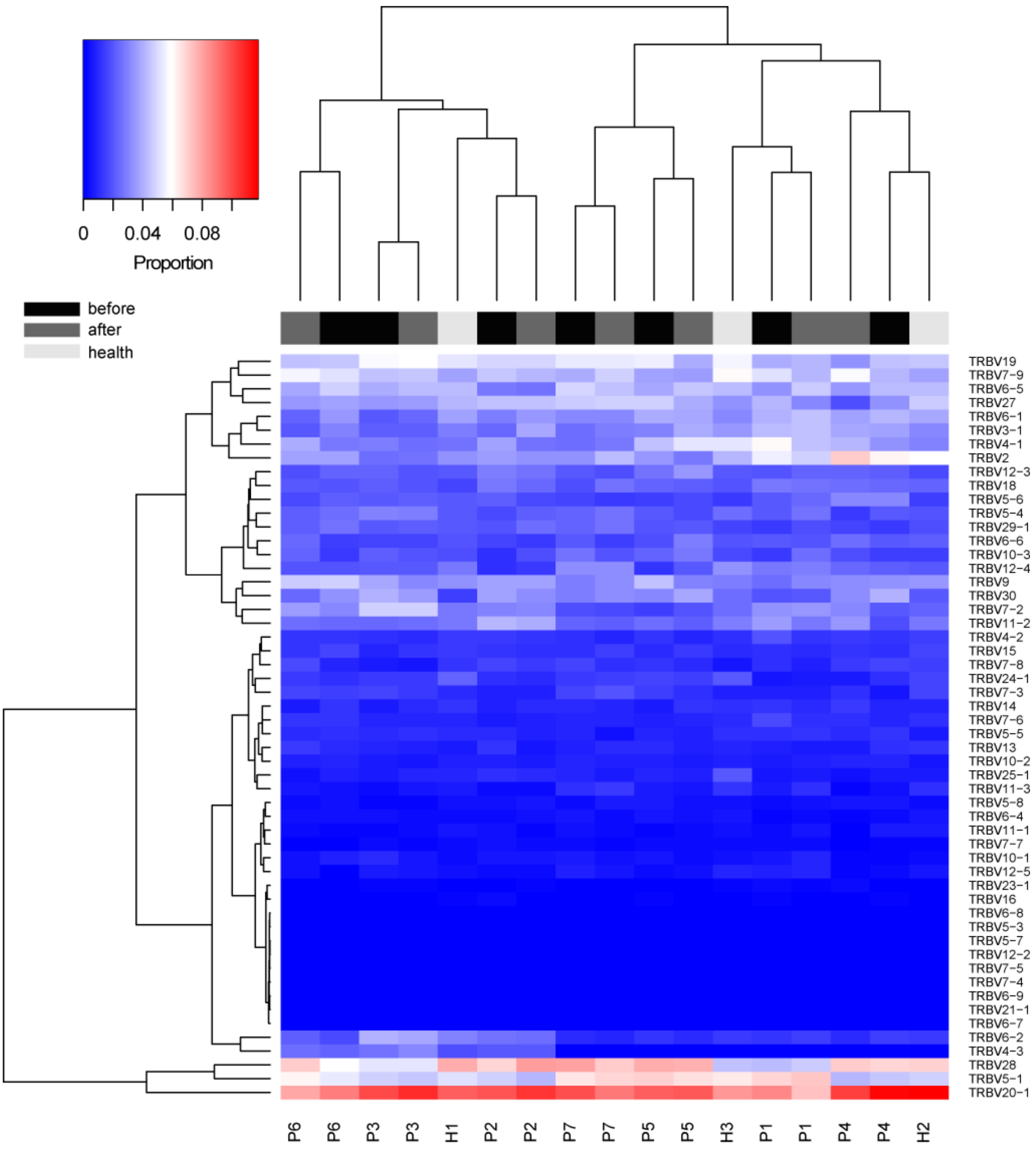

Supplementary Figure 23. TRBV gene usage in the TCR repertoires. Heat map represents proportion of TRBV genes across samples. Source data are provided in the Source Data file.

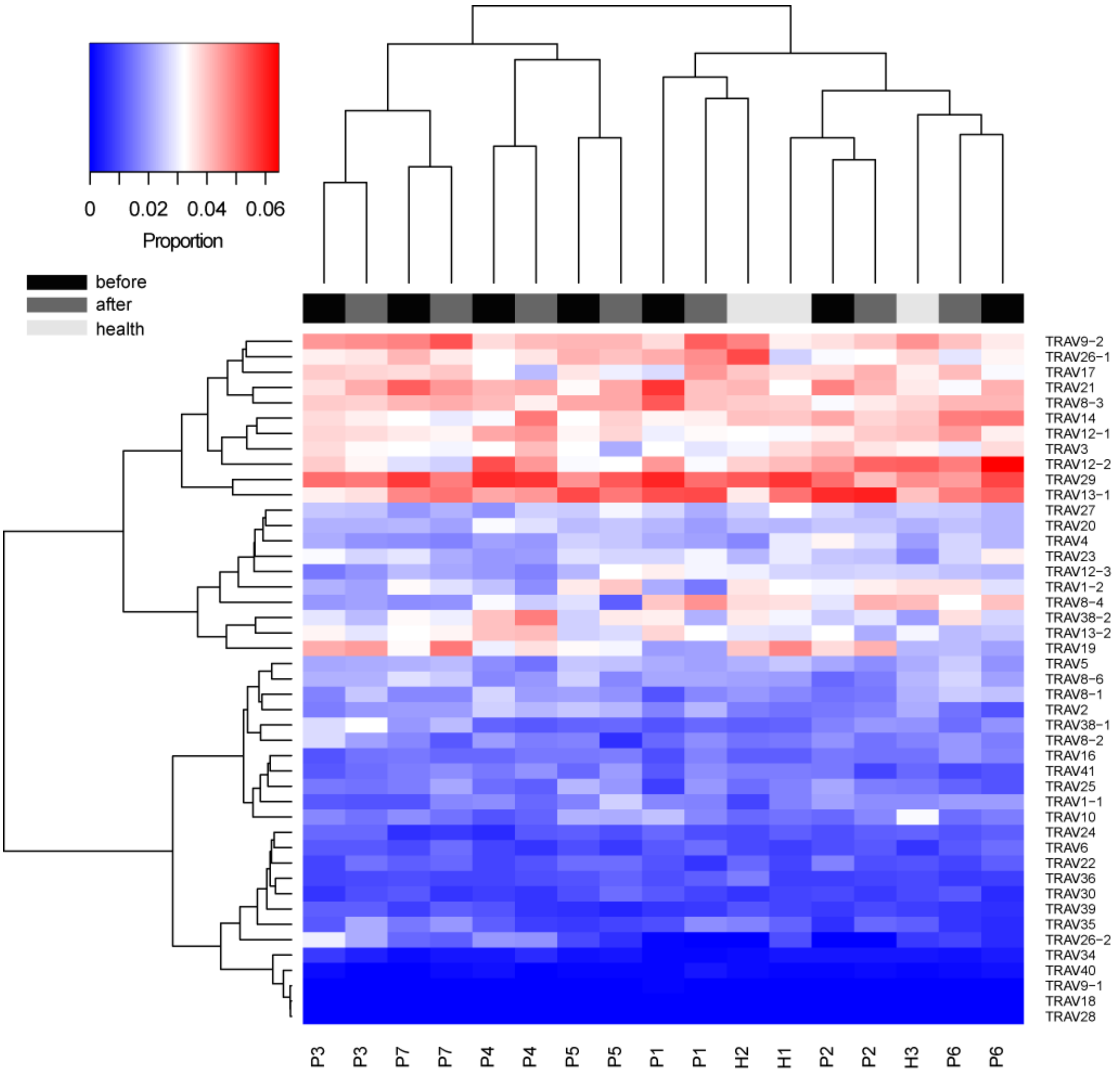

Supplementary Figure 24. TRAV gene usage in the TCR repertoires. Heat map represents proportion of TRAV genes across samples. Source data are provided in the Source Data file.

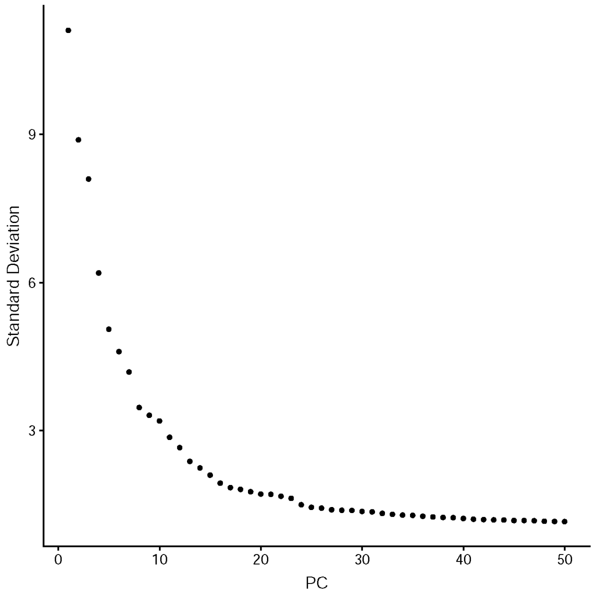

Supplementary Figure 25. Scree plot of PCA. The top 30 principal components (PCs) account for ~95% of the total variance.

Supplementary Table 1. Clinical features of recruited donors.

	**P1**	**P2**	**P3**	**P4**	**P5**	**P6**	**P7**	**H1**	**H2**	**H3**
Age (years)	1.6	5.4	3.3	2.1	1.9	4.7	2	1.7	3	5
Sex (male/female)	F	F	F	M	M	M	M	M	F	F
Fever (days)	7	7	9	7	6	6	7			
Conjunctivitis (yes/no)	Y	Y	Y	N	Y	Y	Y			
Oral changes (yes/no)	Y	N	Y	Y	Y	Y	Y			
Extremity changes (yes/no)	Y	Y	N	Y	N	N	Y			
Rash (yes/no)	Y	Y	N	Y	Y	Y	Y			
Cervical lymphadenopathy (yes/no)	Y	Y	Y	Y	Y	Y	Y			
Complete KD (yes/no)	Y	Y	N	Y	Y	Y	Y			
IVIG responsive (yes/no)	Y	Y	Y	Y	Y	Y	Y			
CAL (yes/no)	N	N	N	N	N	N	N			

Supplementary Table 2. Routine blood test of KD patients before and after IVIG therapy.

	**P1**	**P1**	**P2**	**P2**	**P3**	**P3**	**P4**	**P4**	**P5**	**P5**	**P6**	**P6**	**P7**	**P7**
IVIG therapy	before	after	before	after	before	after	before	after	before	before	after	after	before	after
White blood cell count (10^9/L)	26.04	8.9	6.15	4.68	8.98	4.6	19.4	8.07	18.74	7.25	17.65	7.8	11.35	6.72
Neutrophil count (10^9/L)	19.82	2.79	4.45	2.98	5.93	1.72	14.86	2.22	10.53	2.54	12.94	2.82	7.26	0.41
Lymphocyte count (10^9/L)	4.04	3.54	1.45	1.44	2.38	2.42	2.99	4.54	6.9	4.01	3.11	3.36	3.17	5.12
Monocyte count (10^9/L)	0.57	1.36	0.23	0.14	0.49	0.21	0.33	0.42	0.99	0.39	0.72	0.56	0.54	0.47
Red blood cell count (10^12/L)	4.23	3.97	4.28	3.97	3.67	3.5	3.59	3.41	4.17	3.92	3.9	4.28	4.35	3.54
Platelet count (10^9/L)	469	607	193	293	301	288	291	314	493	658	216	285	186	248
C-reactive protein (mg/L)	170	63	59	42	91	60	120	54	20	19	76	35	31	28

Supplementary Table 3. Quantity and viability of cells loaded onto 10x Genomics.

Donor	IVIG therapy	Cell concentration (cells/μl)	Volume (μl)	Cell viability
P1	before	780	19	0.9
P1	after	1055	11.4	0.95
P2	before	1595	10.3	0.98
P2	after	1035	11.6	0.98
P3	before	950	12.7	0.99
P3	after	500	24	0.98
P4	before	845	14.2	0.93
P4	after	995	12.1	0.98
P5	before	340	32.4	0.95
P5	after	1105	10	0.98
P6	before	510	21.4	0.971
P6	after	835	13.2	0.95
P7	before	955	11.5	0.96
P7	after	630	17.3	0.99
H1	health	1125	10.7	0.98
H2	health	1270	9.4	0.98
H3	health	860	13.9	0.98

Supplementary Table 4. Summary of single-cell RNA sequencing data.

Donor	IVIG therapy	Number of cells	Mean reads per cell	Median UMI counts per cell	Total genes detected	Median genes per cell	Number of cells passing QC
P1	before	6136	54999	1480	17947	624	4083
P1	after	7328	68558	4816	20257	1720	6042
P2	before	6133	57371	3377	18898	1226	5071
P2	after	9910	36008	4052	19548	1415	6885
P3	before	6258	58524	4157	19094	1409	5275
P3	after	8867	39405	5045	19059	1485	7640
P4	before	5794	61228	4372	19627	1324	4890
P4	after	10394	31982	1144	18801	498	3307
P5	before	8790	40311	3274	19379	1285	7911
P5	after	10588	29494	2128	18897	872	5440
P6	before	4292	65307	3722	18764	1269	3374
P6	after	5715	52087	1043	18199	488	2060
P7	before	4047	63971	3727	18515	1312	3469
P7	after	5232	43487	4230	18702	1429	4851
H1	health	5697	65080	3402	18901	1263	5265
H2	health	5080	82405	3265	18913	1229	4382
H3	health	5471	59770	3408	18981	1336	5041

Supplementary Table 5. Summary of single-cell BCR sequencing data.

Donor	IVIG therapy	Number of cells	Mean reads per cell	Number of cells with clonotypes	Number of clonotypes	Number of cells with productive paired chains
P1	before	3242	9958	3230	2977	2546
P1	after	904	27186	903	844	821
P2	before	2180	7805	2170	2129	1873
P2	after	1048	34832	1048	936	986
P3	before	1689	23870	1685	1635	1602
P3	after	976	31256	975	925	945
P4	before	2695	10666	2681	2596	2290
P4	after	7477	3724	7477	1102	954
P5	before	3809	8461	3809	3731	3660
P5	after	8488	3841	8488	1929	948
P6	before	712	64025	712	615	602
P6	after	8108	4981	8108	978	504
P7	before	979	42509	979	955	931
P7	after	1016	38468	1016	989	980
H1	health	1525	21452	1518	1437	1446
H2	health	1313	15319	1305	1254	1231
H3	health	799	33742	792	780	725

Supplementary Table 6. Summary of single-cell TCR sequencing data.

Donor	IVIG therapy	Number of cells	Mean reads per cell	Number of cells with clonotypes	Number of clonotypes	Number of cells with productive paired chains
P1	before	2635	12020	2354	2342	1217
P1	after	3810	6409	3323	3295	2912
P2	before	3597	5542	3284	3162	2594
P2	after	6517	6205	6352	5919	5648
P3	before	3740	22339	3539	3508	3077
P3	after	5028	14485	4835	4792	4400
P4	before	3758	7372	3284	3273	2303
P4	after	3282	7738	3187	3125	2601
P5	before	3276	12103	3276	3244	2837
P5	after	4235	7797	4235	4128	2916
P6	before	1707	27963	1707	1693	1425
P6	after	3163	13532	3163	2966	1924
P7	before	3618	13116	3618	3573	2685
P7	after	3712	11682	3711	3648	3331
H1	health	4472	7107	4087	3658	3351
H2	health	3319	13554	3016	2936	2390
H3	health	3757	8035	3285	3055	2845
