# Supplementary material for: Single-cell RNA sequencing of peripheral blood mononuclear cells from acute Kawasaki disease patients
Source: Nat Commun. 2021 Sep 14;12:5444. doi: 10.1038/s41467-021-25771-5 (PMC8440575; doi:10.1038/s41467-021-25771-5)
Supplement: Supplementary file 3 — Description of Additional Supplementary Files [file 41467_2021_25771_MOESM3_ESM.pdf]

Description of Additional Supplementary Files

File Name: Supplementary Data 1
Description: Differential expression analysis for each cell compartment.

File Name: Supplementary Data 2
Description: Over-representation analysis on gene sets for each cell compartment.
