# Supplementary material for: Single-cell RNA sequencing of peripheral blood mononuclear cells from acute Kawasaki disease patients
Source: Nat Commun. 2021 Sep 14;12:5444. doi: 10.1038/s41467-021-25771-5 (PMC8440575; doi:10.1038/s41467-021-25771-5)
Supplement: Supplementary file 6 — Reporting Summary [file 41467_2021_25771_MOESM6_ESM.pdf]

nature research

Corresponding author(s): Zhen Wang, Yixue Li, Min Huang

Last updated by author(s): Aug 17, 2021

Reporting Summary

Nature Research wishes to improve the reproducibility of the work that we publish. This form provides structure for consistency and transparency in reporting. For further information on Nature Research policies, see our Editorial Policies and the Editorial Policy Checklist.

Statistics

For all statistical analyses, confirm that the following items are present in the figure legend, table legend, main text, or Methods section.

n/a	Confirmed	
☐	☒	The exact sample size (*n*) for each experimental group/condition, given as a discrete number and unit of measurement
☐	☒	A statement on whether measurements were taken from distinct samples or whether the same sample was measured repeatedly
☐	☒	The statistical test(s) used AND whether they are one- or two-sided<br>*Only common tests should be described solely by name; describe more complex techniques in the Methods section.*
☒	☐	A description of all covariates tested
☐	☒	A description of any assumptions or corrections, such as tests of normality and adjustment for multiple comparisons
☐	☒	A full description of the statistical parameters including central tendency (e.g. means) or other basic estimates (e.g. regression coefficient) AND variation (e.g. standard deviation) or associated estimates of uncertainty (e.g. confidence intervals)
☐	☒	For null hypothesis testing, the test statistic (e.g. *F*, *t*, *r*) with confidence intervals, effect sizes, degrees of freedom and *P* value noted<br>*Give P values as exact values whenever suitable.*
☒	☐	For Bayesian analysis, information on the choice of priors and Markov chain Monte Carlo settings
☒	☐	For hierarchical and complex designs, identification of the appropriate level for tests and full reporting of outcomes
☐	☒	Estimates of effect sizes (e.g. Cohen's *d*, Pearson's *r*), indicating how they were calculated

Our web collection on statistics for biologists contains articles on many of the points above.

Software and code

Policy information about availability of computer code

Data collection	Cell Ranger (v3.0.1, 10X Genomics) was used to process scRNA-seq data and generate single-cell expression matrix. Cell Ranger VDJ pipeline was used to assemble single-cell BCRs and TCRs. Flow cytometry data were collected by BD FACSDiva (v8.0).
Data analysis	Seurat (v3.0.2) was used to perform quality control, sample aggregating, dimension reduction, clustering and visualization. SingleR (v1.0.6) was used for automatic cell cluster annotation. DESeq2 (v1.28.1) was used to perform differential expression analysis. clusterProfiler (v3.16.0) was used for function over-representation analysis. GSEA (v4.0.3) was used for gene set enrichment analysis. immunarch (v0.6.5) was used to analyze clonotypes of BCRs and TCRs.

For manuscripts utilizing custom algorithms or software that are central to the research but not yet described in published literature, software must be made available to editors and reviewers. We strongly encourage code deposition in a community repository (e.g. GitHub). See the Nature Research guidelines for submitting code & software for further information.

Data

Policy information about availability of data

All manuscripts must include a data availability statement. This statement should provide the following information, where applicable:

- Accession codes, unique identifiers, or web links for publicly available datasets
- A list of figures that have associated raw data
- A description of any restrictions on data availability

The processed sequencing data generated in this study have been deposited in the Gene Expression Omnibus (GEO) database under accession code GSE168732 (https://www.ncbi.nlm.nih.gov/geo/query/acc.cgi?acc=GSE168732). The raw sequence data generated in this study have been deposited in the National Omics Data Encyclopedia database of Bio-Med Big Data Center, Shanghai Institute of Nutrition and Health, Chinese Academy of Sciences under accession code OEP001162 (https://www.biosino.org/node/). The raw sequence data are available under restricted access for data privacy laws, and access can be obtained by reasonable

request to the corresponding authors. The bulk expression data used in this study are available in the GEO database under accession code GSE73577 (https://www.ncbi.nlm.nih.gov/geo/query/acc.cgi?acc=GSE73577). Source data of Figs. 1-5 and Supplementary Figs. 3, 4, 7, 11, 12, 13, 16, 19, 20, 22, 23, 24 are provided in the Source Data file.

Field-specific reporting

Please select the one below that is the best fit for your research. If you are not sure, read the appropriate sections before making your selection.

- [x] Life sciences
- [ ] Behavioural & social sciences
- [ ] Ecological, evolutionary & environmental sciences

For a reference copy of the document with all sections, see nature.com/documents/nr-reporting-summary-flat.pdf

Life sciences study design

All studies must disclose on these points even when the disclosure is negative.

Sample size	10 donors were subjected to scRNA-seq, including 7 KD patients with every patient sampled twice (before and after IVIG therapy) and 3 healthy controls. Since this is an exploratory study, sample size cannot be pre-determined precisely based on statistical methods, but was chosen with at least three independent biological replicates for each condition. Only results with statistical significance were reported, representing sufficient power under current sample size.
Data exclusions	In scRNA-seq data analysis, cells with total UMI count lower than 2,000 or higher than 60,000 were removed for most samples. For P1 before therapy, a lower cutoff of total UMI count (1,000) was used due to its lower median UMI count per cell. Cells with greater than 5% of sequencing reads aligned to mitochondrial genes were also removed. This is a recommended practice for quality control in scRNA-seq, because low total UMI counts or high mitochondrial UMI counts may represent dying cells, while high total UMI counts may represent doublets.
Replication	Flow cytometric analysis of additional 16 KD patients (before and after IVIG therapy) and 20 healthy controls was performed for replication. All replication attempts were successful.
Randomization	As this is a case/control study and all patients in the KD group were treated with IVIG, randomization was not applicable for group allocation. Within each group, age and sex of individuals were random.
Blinding	Investigators who performed scRNA-seq or flow cytometric analysis for individuals were blinded to group allocation, but blinding was not applicable for investigators who performed comparison between groups.

Reporting for specific materials, systems and methods

We require information from authors about some types of materials, experimental systems and methods used in many studies. Here, indicate whether each material, system or method listed is relevant to your study. If you are not sure if a list item applies to your research, read the appropriate section before selecting a response.

Materials & experimental systems

n/a	Involved in the study	
[ ]	[x]	Antibodies
[x]	[ ]	Eukaryotic cell lines
[x]	[ ]	Palaeontology and archaeology
[x]	[ ]	Animals and other organisms
[ ]	[x]	Human research participants
[x]	[ ]	Clinical data
[x]	[ ]	Dual use research of concern

Methods

n/a	Involved in the study	
[x]	[ ]	ChIP-seq
[ ]	[x]	Flow cytometry
[x]	[ ]	MRI-based neuroimaging

Antibodies

Antibodies used	All antibodies were purchased from BD Biosciences: BD Multitest IMK Kit (Cat# 662965, 1:2 dilution), anti-human CD19-APC-H7 (Cat# 641395, 1:2 dilution), anti-human CD24-PE (Cat# 555428, 1:3 dilution), anti-human CD38-FITC (Cat# 340909, 1:3 dilution), anti-human CD3-V450 (Cat# 560365, 1:2 dilution), anti-human CD8-PerCP (Cat# 652829, 1:5 dilution), anti-human CD4-APC-H7 (Cat# 641398, 1:2 dilution), anti-human CD27-APC (Cat# 558664, 1:10 dilution), anti-human CD45RA-PE-Cy7 (Cat# 560675, 1:2 dilution).
Validation	All antibodies used are commercially available and the validation information can be found on the website of BD Biosciences.

Human research participants

Policy information about studies involving human research participants

Population characteristics	The 23 KD patients were aged 0.4 to 7.2 years old (male: 14, female: 9). The diagnosis of KD was made by using the criteria proposed by the American Heart Association. Most patients met criteria for complete KD except P3 for incomplete KD. They were all IVIG responders and did not develop CALs. The 23 healthy controls were aged 1.2 to 5.5 years old (male: 14, female: 9). They were recruited at routine physical examinations and presented no recent history of fever, infection or immunization.
Recruitment	The donors were recruited from Shanghai Children's Hospital between December 2019 and December 2020. There was a selection bias for complete KD because accurate diagnosis of incomplete KD was often challenging. So this study mainly characterized the immune cell alterations for complete KD.
Ethics oversight	The study was approved by the Ethics Committee of the Shanghai Children's Hospital (IRB Protocol #2019R081). All donors or their legal guardians provided informed consent.

Note that full information on the approval of the study protocol must also be provided in the manuscript.

Flow Cytometry

Plots

Confirm that:

- [x] The axis labels state the marker and fluorochrome used (e.g. CD4-FITC).
- [x] The axis scales are clearly visible. Include numbers along axes only for bottom left plot of group (a 'group' is an analysis of identical markers).
- [x] All plots are contour plots with outliers or pseudocolor plots.
- [x] A numerical value for number of cells or percentage (with statistics) is provided.

Methodology

Sample preparation	EDTA-anticoagulated whole blood was transferred to the laboratory and processed immediately after collection. After 15 min incubation for staining in the dark at room temperature, red blood cells were lysed with 1 ml RBC lysis buffer for 10 min. Then the cells were washed with 1 ml PBS for three times. About 1e4 cells were collected in the flow cytometer.
Instrument	BD FACSCanto II
Software	BD FACSDiva
Cell population abundance	No cell populations were sorted.
Gating strategy	Firstly, intact cells were gated based on SSC and FSC signals. B cells were gated with CD19+, within which plasma cells were gated with CD38++ and CD24-. T cells were gated with CD3+, in which CD8 T cells were gated with CD8+CD4-. Naive and effector memory CD8 T cells were then gated with CD27+CD45RA+ and CD27-, respectively. The gating strategy was illustrated in Supplementary Fig. 10 and Fig. 18.

- [x] Tick this box to confirm that a figure exemplifying the gating strategy is provided in the Supplementary Information.
